# Electrical Transport in Iron Phosphate-Based Glass-(Ceramics): Insights into the Role of B_2_O_3_ and HfO_2_ from Model-Free Scaling Procedures

**DOI:** 10.3390/nano12040639

**Published:** 2022-02-14

**Authors:** Arijeta Bafti, Shiro Kubuki, Hüseyin Ertap, Mustafa Yüksek, Mevlüt Karabulut, Andrea Moguš-Milanković, Luka Pavić

**Affiliations:** 1 Faculty of Chemical Engineering and Technology, University of Zagreb, Marulićev Trg 19, 10000 Zagreb, Croatia; abafti@fkit.hr; 2 Department of Chemistry, Graduate School of Science, Tokyo Metropolitan University, 1-1, Minami-Osawa, Tokyo 192-0397, Japan; kubuki@tmu.ac.jp; 3 Department of Physics, Kafkas University, Kars 36100, Turkey; huseyinertap@kafkas.edu.tr; 4 Electrical & Electronics Department, Faculty of Engineering and Natural Sciences, İskenderun Technical University, İskenderun 31200, Turkey; mustafa.yuksek@iste.edu.tr; 5 Department of Physics, Gebze Technical University, Gebze 41400, Turkey; mevlutk@gtu.edu.tr; 6 Ruđer Bošković Institute, 10000 Zagreb, Croatia; mogus@irb.hr

**Keywords:** iron phosphate glass, small polaron hopping, impedance spectroscopy, scaling procedures, model-free, conductivity and permittivity spectra

## Abstract

In this work, we report the effect of the addition of modifiers and network formers on the polaronic transport in iron phosphate glasses (IPG) in two systems of HfO_2_–B_2_O_3_–Fe_2_O_3_–P_2_O_5_, to which up to 8 mol% boron and hafnium are added. The addition of oxides significantly changes the Fe^2+^/Fe_total_ ratio, thus directly affecting the polaron number density and consequently controlling DC conductivity trends for both series studied by impedance spectroscopy. Moreover, we found that short-range polaron dynamics are also under the influence of structural changes. Therefore, we have studied them in detail using model-free scaling procedures, Summerfield and Sidebottom scaling. An attempt to construct a super-master curve revealed that in addition to change in polaron number density, also the polaron hopping lengths change, and Sidebottom scaling yields a super-master curve. The spatial extent of the localized motion of polarons is correlated with polaron number density and two distinct regions are observed. A strong increase in the spatial extent of the polaron hopping jump could be related either to the structural changes due to the addition of HfO_2_ and B_2_O_3_ and their effects on the formation of polarons or to an inherent property of polaron transport in IP glasses with low polaron number density.

## 1. Introduction

The great compositional flexibility of phosphate glasses (PG) along with properties such as low melting and transition temperatures, high thermal expansions coefficients, and ultraviolet transmission, and electrical conductivity, makes this family of glasses excellent candidates for the study of a variety of applications [1,2,3,4,5,6,7]. Modification of PG expands their applicability as their properties are altered. Generally, the existence of slightly hydrated P–O–P bridges leads to their corrosion, which is triggered by water molecules [8]. Replacing these P–O-P–bonds with more moisture-resistant bonds such as P–O–Fe or even P–O–Al with the addition of Fe_2_O_3_ and Al_2_O_3_ could improve the glass properties [9,10,11,12,13]. Therefore, the incorporation of modifier ions into phosphate glasses is a step forward in optimizing the target properties. Iron, as a transition metal (TM), can occur in phosphate glasses as both, Fe^2+^ and Fe^3+^ [14,15], which strongly depends on the preparation conditions such as melting temperature, time, quenching method, composition, and affects the glass properties [16,17]. It has been shown that increasing the iron content in iron phosphate glasses (IPG) leads to an increase in the Fe^2+^ content, as well as a systematic evolution of the phosphate network (from meta- to orthophosphate structure) which further leads to a strengthening of the glass network [18]. The best chemical durability for binary IPG was found for an approximate composition of 40Fe_2_O_3_–60P_2_O_5_ (mol%), melted under oxidation conditions [11,16,17]. Controlled crystallization (i.e., fabrication of glass-ceramics) of glasses in general, could improve the electrical and magnetic properties [18,19,20,21,22,23,24,25]. It is well known that nanomaterials usually have different properties than the corresponding bulk materials. The formation of nanocrystals in an amorphous environment leads to superior properties which depend on the nature, size, and distribution of crystalline phases in the glassy or other amorphous matrices [26,27,28].

The study of electrical transport in binary IPG began in 1965 [29], and since then the interest in this glass family has been growing steadily [30]. The reason for the continued study of electrical properties could be the fact that the addition of various oxides affects electrical properties. For example, the presence of alkali oxide leads to mixed ion-polaron conductivity [13,30,31,32], while the presence of additional TMO (e.g., MoO_3_, V_2_O_5_, WO_3_) causes a mixed TMO effect [31,33]. Moreover, the addition of rare earth metals in IP glasses leads to materials that can be used in lasers and optoelectronic devices [34,35]. In particular, the addition of other oxides can affect the glass structure as modifiers or network formers, or even influence the redox properties of iron during melting and consequently have a prevailing effect on electrical conductivity properties.

In general, IPG exhibits electronic conduction with a polaronic conduction mechanism [30,36,37,38,39,40]. Thus, conduction occurs through thermally activated hopping of small polarons (from Fe^2+^ to Fe^3+^). The polaron transport strongly depends on the final iron oxide content and the fraction of TM ions in different valence states, as well as the average distance between them. The electrical conductivity of these glasses can vary by several orders of magnitude depending on the aforementioned parameters.

The IPGs are also known to be candidates for the vitrification of certain nuclear wastes. Due to the difficulty of working with real nuclear waste (i.e., Pu^2+^, Pu^4+^), various rare earths are used as actinide surrogates such as Ce^4+^, Hf^4+^, Nd^3+^, etc. The addition of rare elements to borosilicate and iron phosphate glasses has been shown to affect the glass properties [41,42,43,44,45]. Moreover, the incorporation of boron ions into various PGs leads to an increase in thermal and radiation stability and long-term storage due to its high neutron absorption cross-section [46,47,48,49,50]. The addition of modifiers and/or network-forming oxides [44,45,46,47,48,49,50] influences also the glass structure, which in turn has a significant effect on the transport properties [51,52,53]. It has been shown that in iron borophosphate (IBP) glasses [50,51] DC conductivity is directly affected by the polaron number density which is determined by the total amount of Fe_2_O_3_ and is not directly related to B_2_O_3_. On the other hand, the addition of HfO_2_ [44], and CeO_2_ [45] to I(B)P-based glasses, leads to an increase of the Fe^2+^ content up to 60%, which was of great interest for the study of the polaronic conduction in these glass-(ceramics) [53]. On the other hand, the dependence of the values for the spatial extent of the localized polaron motions on the polaron number density for IBP glasses [51,52] complements well the dependence observed for IP glasses with HfO_2_ and CeO_2_ with larger polaron number density [53].

This paper presents the relationship between structural and electrical properties in IPG glass-(ceramics) where up to 8 mol% boron and hafnium oxide are gradually added to 40Fe_2_O_3_–60P_2_O_5_ (IP) base glass. For this purpose, two series of samples were prepared in which the simultaneous addition of B_2_O_3_ and HfO_2_ at the expense of (i) Fe_2_O_3_ (F–series), and (ii) both Fe_2_O_3_ and P_2_O_5_ (S–series) to keep the Fe/P ratio constant (0.67), involving smaller changes in overall Fe_2_O_3_ amount. This work aims to analyze in detail and discuss the variation of the electrical properties that could arise due to structural differences in these glass-(ceramics) and to investigate the role of the structure (simultaneous addition of modifier and network-forming oxide) along with parameters obtained that determine the resulting polaronic transport.

## 2. Materials and Methods

The batch composition of two series of glasses labeled F and S are selected for this study. Series F glasses have the nominal composition *x*HfO_2_–*y*B_2_O_3_–(40 − (*x* + *y*))Fe_2_O_3_–60P_2_O_5_ (*x* = 2–8; *y* = 2–6, mol%) while the composition of the glasses of series S is *x*HfO_2_–*y*B_2_O_3_–(100 − (*x* + *y*))[40Fe_2_O_3_–60P_2_O_5_] (*x*,*y* = 2–8, mol%). Glasses are prepared by melting homogeneous mixtures of reagent grade chemicals (HfO_2_–B_2_O_3_–Fe_2_O_3_–P_2_O_5_) in appropriate amounts in high-density alumina crucibles at 1200–1250 °C in the air for 1–2 h. It is known that P_2_O_5_ and B_2_O_3_ loss during melting is negligible for iron phosphate/borophosphate glasses. The experimental details of the preparation, as well as the structural characterization of these glasses, can be found in reference [44].

In the F–series, Fe_2_O_3_ is replaced by HfO_2_ and B_2_O_3_, while in the S–series, HfO_2_ and B_2_O_3_ are simultaneously replaced by Fe_2_O_3_ and P_2_O_5_. The Fe/P ratio in the initial mixture is kept constant (0.67) for the S series. The samples are labeled according to the amount of boron and hafnium oxide in the batch. For instance, the glass F–B2Hf2 belongs to the F series and contains 2 mol% each of B_2_O_3_ and HfO_2_. The PXRD results of these IPG-based glasses showed the precipitation of the crystalline phase HfP_2_O_7_ in the glass matrix, but this did not affect the chemical stability of the obtained partially crystallized glasses. A similar effect, namely surface crystallization, was also observed in I(B)P glasses [46], and as reported in [51,52] the electrical properties showed only the contribution of the bulk to the overall conductivity process, without any influence of the crystalline phase(s) or grain boundary. The batch compositions along with the number of iron ions per volume, *N*, calculated from the glass composition and density, and the fraction of ferrous ions, C = Fe^2+^/Fe_tot_ ratio, determined by Mössbauer spectroscopy, are given for all these glasses in Table 1 and Table 2.

^57^Fe Mössbauer spectra were collected in order to characterize the valence state of iron ions and to determine relative amounts of Fe^2+^ and Fe^3+^ in studied glasses. Measurements were performed at room temperature (RT) using a constant acceleration method with a source of ^57^Co(Rh) having the activity of 925 MBq. For the measurement, a well-pulverized sample weighing 40 mg was homogeneously dispersed on the transparent adhesive tape in the diameter of 10 mm. The obtained spectra are presented in the Supporting Information (see Figure S1) and analyzed in Lorentzian fitting by using Mösswinn 3.0i XP. Isomer shifts are given relative to α-Fe at RT.

Electrical and dielectric properties were studied by impedance spectroscopy. Before performing measurements, annealed bars were cut into disks. For the contacts, thin gold electrodes were sputtered onto both sides of 1 mm-thick sample disks using a sputter coater SC7620, Quorum Technologies (Laughton, UK). Complex impedance was measured using an impedance analyzer (Novocontrol Alpha-AN Dielectric Spectrometer, Novocontrol Technologies GmbH and Co. KG, Montabaur, Germany) over a wide frequency and temperature range, from 0.01 Hz to 1 MHz at temperatures between 30 °C and 240 °C. The temperature was controlled to ±0.2 °C. The typical complex impedance plot consists of a single semicircle with the centers below the real axis. The equivalent circuit that represents each such depressed semicircle, with the center below the real axis is a parallel combination of a resistor (*R*) and constant-phase element (*CPE*). The *CPE* is an empirical impedance function of the type:

$$\;Z^{*}{}_{CPE}=\frac{1}{A{\left(i\omega \right)}^{\alpha }}$$

where A and α are constants. Experimental data were analyzed by equivalent circuit modeling using the complex non-linear least-square (CNLLSQ) fitting procedure and the corresponding parameters were determined with software [54]. This procedure is based on the fitting of experimental impedance spectra to an appropriate equivalent circuit model.

## 3. Results and Discussion

### 3.1. Impedance Spectra and Direct Current (DC) Conductivity

**Figure 1:**
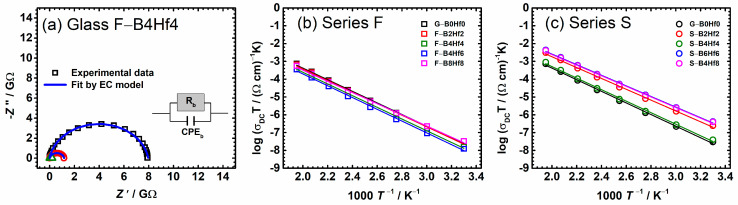
(**a**) Complex impedance plots at different temperatures and (**b**,**c**) Arrhenius plots of direct current (DC) conductivity (log(*σ*_DC_*T*) vs. 1000/*T*) for individual samples from (**b**) F– and (**c**) S–glass series. The corresponding equivalent circuit in (**a**) used for fitting the data is shown in the inset. Open squares denote experimental values; a solid black line corresponds to the best fit. Solid lines in (**b**,**c**) represent the least-square linear fits to experimental data. The error bars are, at most, of the order of the symbol size.

The impedance spectra at different temperatures for F–B4Hf4 sample and their corresponding equivalent electric circuit (EEC) are shown in Figure 1a. For all studied samples, the impedance spectrum contains a single semicircle related to the bulk effects (see Figure S2 in the Supplementary Materials). This is characteristic of electronic conductors [30,55,56], so a simplified single parallel RC element in the equivalent circuit is used for modeling. Preferably, such a semicircular arc passes through the origin of the complex plot and leads to a low-frequency intersection point on the real axis of the complex plot, corresponding to the resistance, *R*, of the sample. From the resistance values obtained from the fitting procedures, *R*, and the electrode dimensions (*d* sample thickness, and *A* electrode area) the DC conductivity is calculated, *σ*_DC_ = *d*/(*R* × *A*), and is listed in Table 2. It should be noted here that the structural study [44] confirms partial crystallization for our glasses. However, the presence of the crystalline HfP_2_O_7_ phase in the amorphous matrix and its influence on the electrical processes are not detected by impedance spectroscopy (IS). In the case of classical glass-ceramics, more than one semicircle is usually present in the IS spectra, which is due to the formation of crystalline phases in a glassy matrix [22,23,24]. Therefore, we conclude that the amount and type of crystalline phase in all glass-(ceramics) from this study, even though confirmed by PXRD, is not significant enough to be detected with IS and, more importantly, does not affect the dominant bulk electronic contribution to overall electrical transport.

Figure 1a shows that the temperature increase results in a decrease of semicircles size and the corresponding resistance decreases, while the DC conductivity calculated from the equivalent circuit modeling increases. Austin and Mott proposed a detailed theoretical approach to the conduction process and activation energy of transition metal oxide (TMO) glasses with their model [24,25]. As is well known, conduction in IPG-based glasses at temperatures above RT is considered as phonon-assisted small polaron hopping (SPH) between local neighboring sites [30,36,37,38,57,58] and the DC conductivity exhibits an Arrhenius temperature dependence with characteristic activation energy. The temperature dependence of DC conductivity, *σ*_DC_, is usually expressed by the Austin–Mott relation [36,37,57]:

(1) $$\sigma_{\mathrm{DC}}T=\sigma_{0}^{*}{}^{\left(-E_{\mathrm{DC}}/k_{B}T\right)}$$

where *σ*_DC_ is DC conductivity, *σ*_0_* is the pre-exponential factor, *E*_DC_ is the activation energy for the DC conductivity, k_*B*_ the Boltzmann constant, and *T* the temperature (K). The pre-exponential factor, *σ*_0_*, contains important parameters for polaronic transport according to the relation:

(2) $$\sigma_{0}^{*}=(C\left(1-C\right)\nu_{ph}e^{2}/Rk_{B})exp\left(-2\alpha R\right)$$

where *α* is the rate of wave function decay, *C* is the fraction of Fe^2+^ ions to total iron content (Fe^2+^/Fe_tot_), and *R* is the average hopping distance between transition metal ions (*R* = *N*^(−1/3)), *ν_ph_* is the phonon frequency (≈10^12^–10^13^ Hz), e is the electronic charge. For the adiabatic hopping conduction, the tunneling term *exp*(−2*αR*) in Equation (2) is approximately 1, by what *αR* becomes negligible. The DC conductivity variation with 1000/*T* for all studied compositions is shown in Figure 1b,c. The activation energy, *E*_DC_, for each sample is calculated from the slope of log *σ*_DC_*T* vs. 1000/*T*. Calculated values along with the values of DC conductivity at 30 °C and pre-exponential factor are given in Table 2. The values of activation energy range from 56.9 to 63.3 kJ/mol, while the DC conductivity at 30 °C is between 1.35 × 10^−9^ (Ω cm)^−1^ and 4.07 × 10^−11^ (Ω cm)^−1^, both of which agree well with the values for various IPG-based glasses from the literature [30,39,51,53,56,59,60].

**Figure 2:**
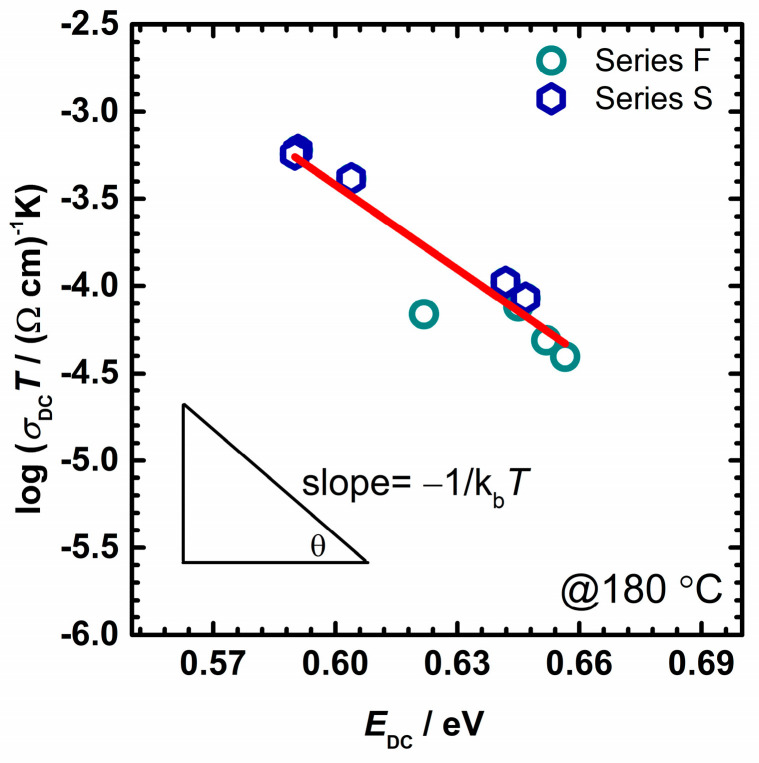
Effect of activation energy, *E*_DC_, on DC conductivity, *σ*_DC_*T*, for studied F– and S–glass series. Each point represents an individual sample. The solid line represents the least-squares linear fit to experimental data. The error bars are, at most, of the order of the symbol size.

Stepping forward in the analysis of the electrical transport in these glass-(ceramics), we focus on the changes in the pre-exponential factor and other parameters, see Table 2. According to the Austin–Mott theory of SPH, the conduction can be characterized by either adiabatic or non-adiabatic hopping. In adiabatic hopping, the electron is relaxed at all times and can respond rapidly to the lattice displacement, while in non-adiabatic hopping there is a small chance for electron tunneling [36,37,57]. Thus, in adiabatic hopping conduction *αR* in Equation (2) becomes negligible. Based on the data available, it was expected that in the case of IPG-based glasses, the non-adiabatic hopping model is more suited for describing polaron transport [51]. To verify whether the nature of the hopping conduction in the samples of this study is adiabatic or non-adiabatic, the plot log(*σ*_DC_*T*) vs. activation energy, *E*_DC,_ at a fixed experimental temperature *T* = 180 °C is plotted and presented in Figure 2. The observed slope should be equal to −1/k_*B*_*T* based on the DC conductivity in the adiabatic regime given by Equation (2). If the temperature obtained from the slope differs from the experimental temperature, the process is considered non-adiabatic. The temperature we obtained from the slope is equal to *T* = 40 °C, which is very different from the chosen temperature of 180 °C. This indicates the non-adiabatic hopping of small polaron and strong electron-phonon coupling in our studied samples and is supported by previously reported literature data on similar IP-based glasses [51,53,61,62].

The values of the tunneling factor, α, for each glass are calculated from the obtained values of pre-exponential factors, the fraction of Fe^2+^ ions (*C* values), and based on the assumption that the size distribution is random, see Table 2. The calculated values are between 0.37 and 0.58 Å^−1^ which is in good correlation with those for similar IP-based glasses reported in the literature [37,60,63,64].

**Figure 3:**
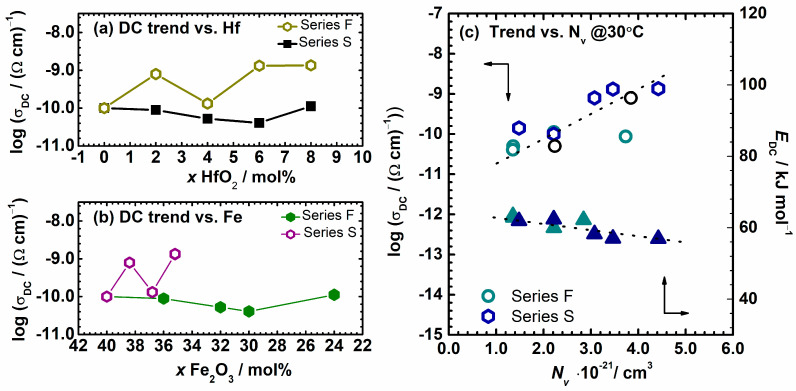
DC conductivity at 30 °C and activation energy for DC conductivity, *E*_DC_, as a function of (**a**) HfO_2,_ (**b**) Fe_2_O_3,_ and (**c**) the number density of polarons, *N*_v_, for both series. Additionally, two black circle symbols in (**c**) are the experimental data for two samples from this study, with *N*_v_ calculated based on Mössbauer data reported in reference [44]. The error bars are, at most, of the order of the symbol size. Lines are drawn as guides for the eye.

The dependence of DC conductivity for both series upon HfO_2_ and Fe_2_O_3_ content is shown in Figure 3a,b. Initially, the two series show completely different behavior concerning each other, without any influence on the type of the content (Fe_2_O_3_ or HfO_2_). The DC conductivity for the F–series varies from 1.00 × 10^−10^ to 4.07 × 10^−11^ (Ω cm)^−1^ at 30 °C with the addition of HfO_2_ content up to 6 mol%, and increases for sample F–B8Hf8 reaching a value of 1.12 × 10^−10^ (Ω cm)^−1^. Otherwise, the S–series shows an opposite behavior, and the DC conductivity increases by almost an order of magnitude with the addition of HfO_2_, from 1.00 × 10^−10^ to 1.35 × 10^−9^ (Ω cm)^−1^ for glasses containing 8 mol% HfO_2_, respectively. Interestingly, sample S–B4Hf4 shows a deviation from this trend. It can also be seen that the variation in the amount of Fe_2_O_3_ content is high in the F–series as it decreases from 40 to 24 mol%, whereas for S–series the range for iron content is narrower, between 40 and 35 mol%, because B_2_O_3_ and HfO_2_ are added at the expense of Fe_2_O_3_ and P_2_O_5_ in the composition. The observed trends suggest that the Fe_2_O_3_ content is not the only key parameter behind polaronic transport in these samples. As can be seen from the Mössbauer spectra in Figure S1 and shown in Table 2, although the total Fe_2_O_3_ content decreases in these iron phosphate-based glass-(ceramics), the Fe^2+^ concentration changes from 0.16 to 0.58. Based on this observation, we analyzed the polaron number density parameter and its changes with composition and conductivity trends in more detail.

It should be noted here that the polaron number density depends not only on the total amount of Fe_2_O_3_ but also on the fraction of Fe^2+^ and Fe^3+^ ions and is determined by the product of number density of the total iron ions and a fraction of ferrous ions (or Fe^2+^/Fe_tot_ ratio < 0.5). For a ratio value above 0.5, it is determined as a product of the number density of the total iron ions and the fraction of ferric ions [2,38,53]. The calculated values of polaron number density for all samples are given in Table 3. DC conductivity and activation energy for DC conductivity, *E*_DC_, as a function of the number density of polarons, *N*_v_, for both series of samples is shown in Figure 3c. Indeed, for all compositions studied, a nearly linear trend is observed with an increase of a polaron number density. In particular, the DC conductivity increases while the *E*_DC_ decreases. This supports our conclusion that the polaronic transport in these glass-(ceramics) is not only controlled by the total iron oxide content but a crucial influence comes from the concentration of Fe^2+^ ions, which ultimately affects the polaron number density and governs the trend of DC conductivity. Moreover, for two compositions, namely F–B4Hf4 and S–B2Hf2, the *N*_v_ was also calculated based on Fe^2+^ ratio reported in reference [44] which show slightly higher values. Interestingly, data points also lie on an almost linear dependence of the DC conductivity versus polaron number density and confirm our results, see a black symbol in Figure 3c. The only exception could be seen for the glass with Fe^2+^/Fe_tot_ = 0.58 (F–B2Hf2 sample) which shows a value deviating from linearity. In such a ferrous-rich condition an inhomogeneous distribution of ferrous and ferric ions could be present which means that a significant fraction of ferrous ions cannot contribute to the polaron transport [52].

From a previous structural study of these two systems [44], only a slight difference in the cross-linking of the IP network is observed. In both series with the addition of B_2_O_3_ and HfO_2_, an increase in *T*_g_ value was noticed, slightly higher for F–series where the wider variety of overall Fe_2_O_3_ content is present, and only Fe_2_O_3_ is exchanged with Hf and B. This behavior agrees well with a previous study of IBP glasses modified with boron oxide [46], where the addition of B_2_O_3_ increases thermal stability. It seems likely that in compositions studied, small changes in the strength and rigidity of the IP network do not affect the mechanism of polaronic transport. Nevertheless, even though from Mössbauer analysis both Fe^2+^ and Fe^3+^ ions have distorted octahedral coordination in all studied samples similar to many other iron phosphate systems [11,65], their concentration and Fe^2+^/Fe^3+^ ratio are significantly altered by the addition of B_2_O_3_ and HfO_2_ in both series, which again implies having a dominant effect on the polaron number density and consequently on the DC conductivity trend.

Additionally, we shift our attention from DC conductivity and long-range transport features to the frequency-dependent conductivity and examination of the localized motions of charge carriers.

### 3.2. Scaling Features of the Conductivity Spectra

**Figure 4:**
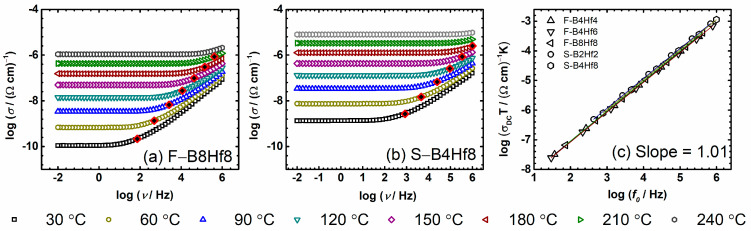
Conductivity spectra for samples (**a**) F–B8Hf8 and (**b**) S–B4Hf4 from series F and S, respectively. Filled squares denote the frequencies of the onset of conductivity dispersion defined at *f*_0_ = 2*σ*_DC_. (**c**) Log-log plot of the two scaling parameters *σ*_DC_*T* and *f*_0_ for several compositions from both series. The straight lines are obtained by least-squares linear fits to experimental data and have a slope of 1.01 ± 0.01 for all studied samples.

The conductivity spectra for F–B8Hf8 and S–B4Hf4 samples from the F– and S–series are shown in Figure 4a. Each isotherm shows universal properties and exhibits two characteristic features: (i) a plateau at low frequencies corresponding to DC conductivity, and (ii) a frequency-dependent region (dispersion) at higher frequencies [66]. The conductivity dispersion, which is visible at lower temperatures, shifts to higher frequencies with temperature increase and gradually leaves the frequency range in our impedance spectroscopy setup. We study the scaling properties of the conductivity over a wide range of frequencies and temperatures in order to gain more information about the mechanism(s) of the electrical transport in these glass-(ceramics). A quite simple, but very useful, means of analyzing the conductivity spectra is based on the use of various scaling techniques. Here we use one of the simplest scaling procedures proposed by Summerfield which uses two experimentally determined parameters: DC conductivity and temperature as scaling parameters [67,68]. Summerfield scaling is expressed by: (*σ*(*ν*,*T*)/(*σ*_DC_*T*)) = *F*(ν/*σ*_DC_*T*) and can be understood as mobility scaling. An indication of it is that the role of temperature is to accelerate or slow down the charge carrier dynamics without influencing the conduction mechanism. If the Summerfield procedure of scaling is valid then both axes in a double log-log plot are scaled by the same factor *σ*_DC_*T*. This is equivalent to scaling the isotherms in such a plot along a line with slope one, which is plotted by marking the onset (initial) frequency value of the conductivity dispersion, *σ*′(*f*_0_) = 2*σ*_DC_, please see Figure 4a,b.

We could make important and indicative observations based on obtained slope values, see Figure 4c. The correlation between the validity of Summerfield scaling and the slope of one has been demonstrated in the literature [53,69]. In our case, for all compositions in this study, the slope is 1.01 ± 0.01, see Figure 4c, suggesting the validity of Summerfield scaling which will be shown and discussed in the following parts of the text.

**Figure 5:**
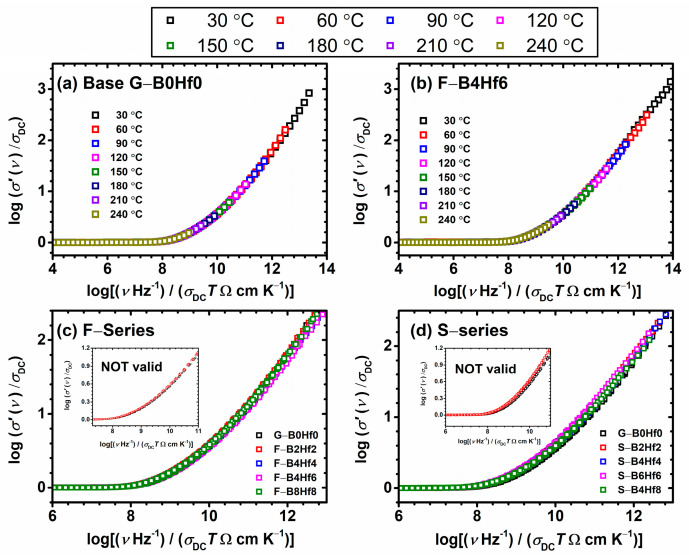
Conductivity spectra scaled according to Summerfield scaling procedure for (**a**) S– and (**b**) F–glass series and (**c**,**d**) construction of super-master curve of the conductivity isotherms using the Summerfield scaling procedure for studied F– and S–glass series. Inset (**c**,**d**): individual master-curves shifted along the *x*-axis to overlap with the reference master-curve of base G–B0Hf0 glass.

Figure 5a,b shows the result of the Summerfield scaling procedure for base glass (G–B0Hf0) and F–B4HF6 sample. One can see that the conductivity isotherms perfectly overlap and form a conductivity master-curve. A similar result is obtained for all other samples from this study and it shows that the time–temperature superposition (TTS) is valid for each composition and the conductivity mechanism does not change with temperature. A behavioral correlation could be undertaken with various oxide glasses that show polaronic [52,53,59,69] or ionic [66,69,70] conductivity, however, mixed ionic-polaronic glasses usually show deviation due to the presence of two different thermally active charge carrier species [69].

**Figure 6:**
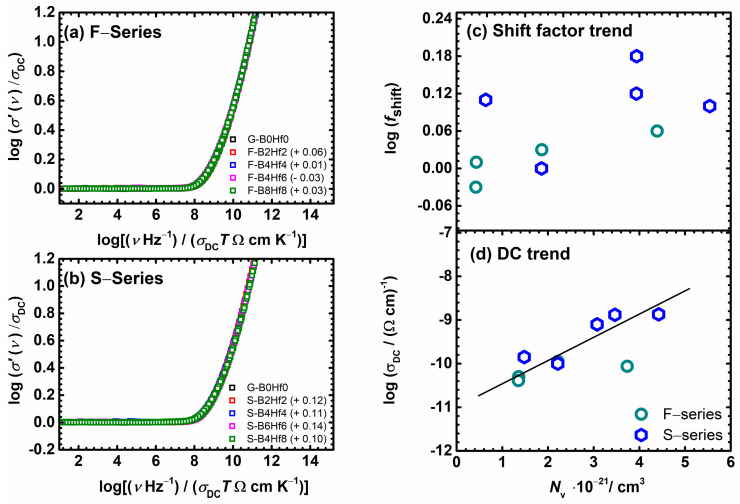
Individual master-curves shifted along the *x*-axis to overlap with the reference master-curve of base glass G–B0Hf0 for (**a**) F– and (**b**) S–glass series. The shift factors in the logarithmic scale, log(*f*_shift_), for all samples are indicated in the legends and listed in Table 3. Plots of (**c**,**d**) log DC conductivity at 30 °C, and log(*f*_shift_) from Summerfield scaling as a function of the number density of charge carriers, *N*_v_, for all samples studied.

It is interesting to investigate the influence of glass composition and structure on the conductivity dispersion, the frequency-dependent part, by applying a superscaling procedure where all individual master-curves within these two series are superimposed. Figure 5c,d shows the result of such superscaling for all the studied samples. As can be seen, the individual master-curves do not overlap perfectly, and a super-master curve could not be obtained, which can be seen in the insets in Figure 5c,d. There are two possible reasons for such a result: either the shape of the conductivity dispersion changes or/and the individual master curves shift along the *x*-axis with the changes in the composition. We shifted the individual master curves along the *x*-axis and tried to create a super-master curve for each series, Figure 6a,b. The master-curve of the base glass (F40) is used as the reference curve in shift calculations. The values of shift required to produce a super-master curve for specific composition are given in the legend of Figure 6a,b. Looking at the magnitude and direction of the shift, small values are found, which leads to perfectly overlapped master-curves when shifted, indicating that the shape of their conductivity dispersion remains the same no matter what the compositional changes.

A shift in the super-master conductivity plot is reported in the literature for different ion-conducting oxide glasses and has been correlated with the alkali content [71,72,73] as well as with changes in the typical length of the hop of the ions with their number density [74]. In our recent paper [53] on IP glasses doped with HfO_2_ and CeO_2_, similar in composition but with widely ranging concentration of ferrous ions (0.23 ≤ Fe^2+^/Fe_tot_ ≤ 0.58), the shift was also observed, but correspondingly in a scattered fashion.

Furthermore, the correlation between log(*f*_shift_) and the changes in the number density of charge carriers, *N*_v_, was studied. The variation of log(*σ*_DC_) and log(*f*_shift_) as a function of *N*_v_ is shown in Figure 6c,d. It can be seen that as charge carrier number density increases, the logarithm of DC conductivity increases nearly linearly, except for the F–B2Hf2 sample, which already exhibited a deviation from linearity probably due to the inhomogeneous distribution of ferrous and ferric ions in the glass matrix [53].

In the top part, Figure 6c, the logarithm of the shift factor for scaling conductivity master curves, log(*f*_shift_), exhibits scattering rather than a linear trend, suggesting that its origin is not entirely related to changes in the polaron number density. This prompts us to consider the alternative scaling procedure proposed by Sidebottom [74,75], which accounts for both changes in number density and the typical hopping distance of the mobile species. To this end, we now examine the information available from permittivity spectra.

### 3.3. Scaling Features of the Permittivity Spectra

**Figure 7:**
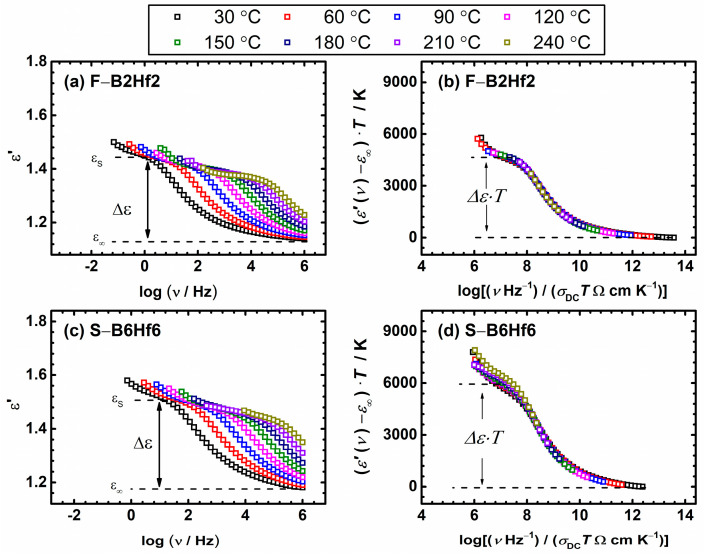
(**a**,**c**) Permittivity spectra at different temperatures and (**b**,**d**) their scaled representation obtained using the Summerfield scaling procedure for the sample F–B2Hf2 and S–B6Hf6.

The complex permittivity ε* (*ν*) = 1/(2π*ν*jC_o_Z*) can be expressed as a complex number:

ε* (*ν*)′ = *ε*′ (*ν*) − j*ε*″ (*ν*) (3)

where *ε*′(*ν*) and *ε*″(*ν*) are the real and imaginary parts of the complex permittivity. The frequency dependence of the real part of the complex permittivity, *ε*′(*ν*), at different temperatures for F–B2Hf2 and S–B6Hf6 samples, is shown in Figure 7a,c. At higher frequencies, the dielectric permittivity approaches a constant value, *ε*′_∞_, resulting from fast polarization processes that occur in the glasses under the applied field [76]. Therefore, the mobile charge carriers cannot rotate sufficiently fast, so their oscillation lags behind this field, decreasing the dielectric permittivity, *ε*′(*ν*). On the other hand, with increasing temperature and decreasing frequency, *ε*′(*ν*) increases and for all the glass-(ceramics) studied, the low-frequency plateau denoted as the value of the low-frequency static permittivity, *ε*_s_, is well-developed, see Figure 7a,c. The observed plateau is related to the polarization effects of long-range hopping of mobile charge carriers concerning the immobile glass matrix in oxide glasses. The magnitude of this polarization, called dielectric strength, is given by Δ*ε*′ = *ε*_s_ − *ε*′_∞_, as proposed by Sidebottom [74,75,77] and represents the rate of permittivity change due to relaxation. For some disordered glasses, where the electrode polarization is significant, the experimental data could not determine the static dielectric constant. However, for the compositions studied in this work, the well-defined low-frequency plateau allows the determination of the permittivity changes and the correlation effects between successful hops. Since *ε*′_∞_ is only weakly temperature-dependent, the dielectric strength for polaronic glasses can be determined directly from the temperature-dependent experimental spectra.

Additionally, in the Summerfield scaling analysis of the relaxation mechanisms, the master curves of the dielectric permittivity spectra for the two compositions mentioned above are obtained since the scaling properties of the conductivities are reflected in the scaling properties of the permittivity data [78]. The *ε*′(*ν*) on the *y*-axis is scaled by the product (*ε*′(*ν*) − *ε*_∞_) *T*, while the frequency *x*-axis is scaled as the product *ν*/*σ*_DC_*T*. The scaled spectra for samples F–B2Hf2 and S–B6Hf6 are shown in Figure 7b,c. As expected from the conductivity data, the application of Summerfield scaling results in a common master curve, and the scaled permittivity data at different temperatures for each sample from this study collapse into a single master curve. From the scaled data, a parameter Δ*ε* × *T* can be extracted, which can be related to the typical spatial extent of localized motions of polarons, <*r*_LOC_^2^(∞)>^1/2^, discussed in the following Section 3.4. In our case, parameter Δ*ε* × *T* is in a range from 3910 to 5742 K and depends on the composition and level of modification of base glass structure with B_2_O_3_ and HfO_2_ (Table 3).

**Figure 8:**
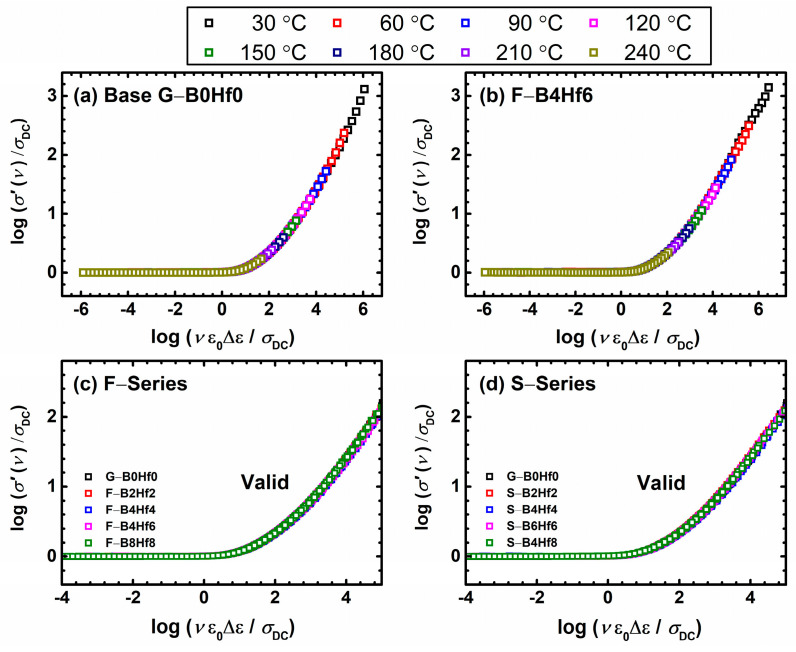
Conductivity spectra scaled according to Sidebottom scaling procedure for (**a**) base G–B0Hf0 and (**b**) F–B4Hf6 samples and (**c**,**d**) construction of super-master curve of the conductivity isotherms using Sidebottom scaling procedure.

As already mentioned above, an alternative scaling procedure for conductivity spectra is proposed by Sidebottom to account for the simultaneous change in typical charge carrier hopping distance with the change in their number density [53,73]. Here, the parameters required from experimental data for scaling the frequency axis are the DC conductivity, the dielectric strength, and the universal constant, the permittivity of free space. This scaling procedure is expressed by the form: (*σ*(*ν*,*T*)/(*σ*_DC_*T*)) = F((*ε*_0_Δ*ε*)/*σ*_DC_ *ν*). Compared to the Summerfield procedure, Sidebottom scaling could be more challenging since for ionic conductive glasses the determination of Δ*ε* is usually hindered by the electrode polarization effect, which is not the case in polaronic glasses. Nonetheless, it can be considered truly universal since it is applicable whenever scaling is possible, i.e., when the shape of the conductivity dispersion does not change with temperature [79]. As far as we know, Sidebottom scaling was first applied to scaling conductivity spectra of polaronic glasses in our recent publications on various IPG–based glasses a few years ago [52,53]. A perfect super-master curve is obtained for all glass-(ceramics) considered in this study, which is not surprising because of the universal background of this scaling. We present this behavior separately for each series, as shown in Figure 8. This feature clearly shows that for our systems, the charge carrier concentration changes along with the typical length of a polaron hop. At the same time, obtaining the super master-curve confirms that the shape of dispersion likewise does not change.

### 3.4. Relevant Length Scales to Electrical Transport

**Figure 9:**
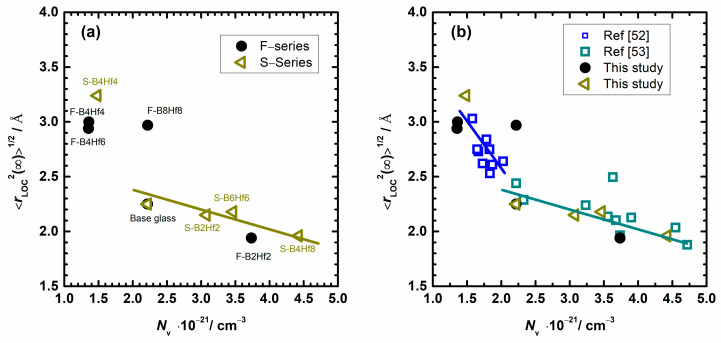
(**a**) The spatial extent of localized motions of polarons, <*r*_LOC_^2^(∞)>^1/2^, as a function of the number density of polarons for studied F and S glass series and (**b**) Values for similar iron phosphate glass (IPG)-based glasses from reference [52,53] are also presented. The lines are drawn as guides for the eye.

With regard to the length scales relevant to electrical transport, it is possible to distinguish different parameters: those calculated based on composition and density such as average distance between iron ions, *R* (see Table 1) and polaron radius, *r*_p_ [80], and those obtained from the experimental data and polaron dynamics. At this point, we benefit from the fact that well-defined permittivity plateaux are visible in our data. To continue in this direction, we decided to estimate the spatial extent of the localized displacement in a model-free approach. In the previous section we showed how for each sample, the scaling of the permittivity spectra yields a parameter Δ*ε T* that can be related to the typical spatial extent of localized motions of polarons, <*r*_LOC_^2^(∞)>^1/2^ by the relation [53,81]:

(4) $${<r_{\mathrm{LOC}}^{2}\left(\infty \right)>}^{1/2}=\;6k_{B}\varepsilon_{0}\Delta \varepsilon T/N_{V}q^{2}$$

where k_*B*_ is the Boltzmann’s constant, *ε*_0_ is the permittivity of free space, and *N*_v_ is the number density of polarons. The obtained values of the extent of the localized motions of polarons are given in Table 3 and shown in Figure 9.

The dependence of <*r*_LOC_^2^(∞)>^1/2^ upon the number density of polarons for studied F– and S–glass series and correlation with literature data is presented in Figure 9. As can be seen, the value of <*r*_LOC_^2^(∞)>^1/2^ for both series varies in the range between 1.87 Å to 3.24 Å. Simultaneously, the polaronic number density changes in the range between 1.35 × 10^−21^ cm^−3^ and 4.42 × 10^−21^ cm^−3^. It is hard to see the trend, but a closer look reveals useful information. The correlation between <*r*_LOC_^2^(∞)>^1/2^ and *N*_v_ for all compositions exhibits two distinct regions. Region 1 contains samples with higher *N*_v_, above ~2.2 × 10^−21^ cm^−3^, whereas the samples with lower *N*_V_ fall into Region 2. It is indicative that the transition between these regions is not only related to *N*_v_, but also to the combination of compositions and parameters which have an impact on <*r*_LOC_^2^(∞)>^1/2^.

The influence of B_2_O_3_ and HfO_2_ on the Fe^2+^ concentration could also be observed. From Table 2 it can be seen that in both series in the first step of modifying IPG glass structure at lower amounts of modifying oxides, boron has the dominant effect on the trend as it decreases the Fe^2+^ concentration with an apparent minimum for compositions F–B4Hf4 and S–B4Hf4 (16% and 18%, respectively). Additional indications of the dominant role of boron on the Fe^2+^ concentration could be seen in sample S–4BHf6 where the content of hafnium oxide increases, however, the Fe^2+^/Fe_total_ ratio is kept low at 18%. In the next step, with simultaneous modification of the glass network, Fe^2+^ concentration increases up to 38% and 36% for samples F–B8Hf8 and S–B4Hf8 and indicates that HfO_2_ takes control and has a positive impact, increasing the Fe^2+^ concentration. A shift from boron oxide to HfO_2_ taking control could further be seen in the fact that for the sample S–B4Hf8, the amount of boron oxide is kept at 4 mol% while HfO_2_ is increased to 8 mol%, which results in a jump of ferrous concentration from 17% to 36%. Also, these results could be compared with Mössbauer’s result for IPG glass with up to 20 mol% of boron oxide [46,52], where it is shown that there is no significant variation in Fe^2+^/Fe_tot_ ratio since the fraction of ferrous ions changes in a narrow range from 0.16 to 0.22. Furthermore, considering <*r*_LOC_^2^(∞)>^1/2^ trends, it should be pointed out that for series S the overall Fe_2_O_3_ content is less under the impact of compositional changes as B_2_O_3_ and HfO_2_ are added at the expense of both F_2_O_3_ and P_2_O_5_ content. This results in a narrower range of iron oxide change in the S–series (40–35 mol%) in comparison to the F–series (40–24 mol%). Both aforementioned parameters affect the general result and values on *N*_v_ and <*r*_LOC_^2^(∞)>^1/2^. For example, one can see that at the shift from the first region to the second, the polaron number density is ~2.2 × 10^−21^ cm^−3^ for two extreme compositions, base glass G–B0Hf0 and F–B8Hf8. This implies that even though the *N*_v_ is similar, modification of IPG structure and addition of B_2_O_3_ and HfO_2_ have an impact on polaron transport. As a result, <*r*_LOC_^2^(∞)>^1/2^ increases suddenly from 2.25 to 3 Å for the sample with a high amount of boron and hafnium oxide.

Returning to two distinct regions in Figure 9a, an interesting feature can be observed. The F–series, where the Fe^2+^ concentration ranges from 0.16 to 0.58, shows a much broader *N*_v_ region. The increase of polaron number density from 2.2 × 10^−21^ cm^−3^ to 4.4 × 10^−21^ cm^−3^ has surprisingly no effect on the <*r*_LOC_^2^(∞)>^1/2^ which is ~3 Å. For the last sample of the F–series, F–B8Hf8, an increase in *N*_V_ is observed which could be attributed to the increase in ferrous concentration due to the positive effect of high HfO_2_ content. At the same time, the DC conductivity trend shows a similar behavior and follows the changes of *N*_v_, see Figure 4. Looking at the changes in <*r*_LOC_^2^(∞)>^1/2^, one can see that, except for the sample F–B2Hf2 [69], the spatial extent of localized motions of polarons is in the low *N*_v_ range (second region) with a value of ~3 Å. Once again, the polaron concentration is higher in S–series, except for S–B4Hf4, and all samples fall into the first region, which is characterized by a high *N*_v_ value. Discussing a general trend, it should be pointed out that the polaron number density increases, whereas <*r*_LOC_^2^(∞)>^1/2^ slightly decreases linearly between 1.9–2.3 Å. However, the jump to 3.2 Å for S–B4Hf4 sample (minimum in Fe^2+^ concentration, impact of the boron oxide) and transitions from the first region to the second one are observed.

At this point, it is useful to examine the results from Figure 9a and Table 3 and compare them with literature data on similar IPG–based glasses, see Figure 9b. First, the observed trend corroborates that the spatial extent of the localized polaron hopping decreases with increasing polaron number density and implies a realistic estimate of the extent of the polaron hop. The magnitude of <*r*_LOC_^2^(∞)>^1/2^ is close to polaron radius, *r*_p_, values calculated using the Bogomolov and Mirilin: *r*_p_ = (1/2) (π/6*N*)^1/3^ relation, where *N* is the total number of iron ions [80]. On the other hand, the *R* parameter calculation based on composition and density shows significantly higher values (see Table 1). The obtained values for <*r*_LOC_^2^(∞)>^1/2^ are in the appropriate range when comparing different IPG systems, and a good correlation can be drawn. It is interesting to see that the compositions from this study cover both regions in Figure 9 (low *N*_v_–higher <*r*_LOC_^2^(∞)>^1/2^ and vice versa) due to wide variation in *N*_v_, which was not the case in our previous studies [51,52,53]. Once again, the change of <*r*_LOC_^2^(∞)>^1/2^ with *N*_V_ is characterized by a larger slope in the lower polaron number density region, see Figure 9b. Taking all this into consideration, it can be concluded that the analysis of the correlation between <*r*_LOC_^2^(∞)>^1/2^ and *N*_V_ allows clear identification of the prevalence and determination of a sharp increase in the spatial extent of the polaron hopping jump, which consequently has a direct impact on the DC conductivity trends in the studied samples. This result could be related either to the structural changes induced by the addition of modifiers or network former oxides and their effects on the formation of polarons or to the inherent property of the polaron transport in IPG with low *N*_v_.

## 4. Conclusions

In this work, we use solid-state impedance spectroscopy to study in detail the electrical properties of iron phosphate glasses in which up to 8 mol% boron and hafnium oxide were added to 40Fe_2_O_3_−60P_2_O_5_ base glass (G–B0Hf0). Two different series of glasses were prepared with simultaneous additions of B_2_O_3_ and HfO_2_ at the expense of Fe_2_O_3_ (F–series), or both Fe_2_O_3_ and P_2_O_5_ (S–series). The observed trends in long-range DC conductivity show that the key parameter behind polaronic transport in these glasses is not only the Fe_2_O_3_ content. The addition of B_2_O_3_ and HfO_2_ significantly alters the Fe^2+^ concentration in both IP glass series, from 0.16 to 0.58, which has a dominant effect on the polaron number density and consequently on the trend of DC conductivity. As a result, a nearly linear trend is observed with an increase in the polaron number density.

Furthermore, we investigated the short-range polaron dynamics by applying a model-free analysis of the conductivity and permittivity spectra of the studied glassy systems and scaling procedures to gain a better insight into the polaron dynamics. Namely, Summerfield and Sidebottom scaling of the conductivity spectra confirmed the validity of the time–temperature superposition principle for all compositions. The construction of the super-master curve reveals an interesting feature. While the shape remains the same, the Summerfield scaling fails, but the Sidebottom one yields a super-master curve for both series. This indicates that in addition to change in polaron number density, also the polaron hopping lengths change. In the next step, we used experimental permittivity spectra to evaluate the spatial extent of the localized motion of polarons. Its correlation with the polaron number density reveals two distinct regions, containing samples with low and high polaron concentrations. Interestingly, the transition between these regions is not only related to the polaron number density, but also to the combination of sample compositions and parameters which have an impact on polaron-localized motions. The detailed analysis allowed clear identification of a sharp increase in the spatial extent of the polaron hopping jump, which consequently has a direct impact on the DC conductivity trends for both series. This feature could either be directly related to the structural changes induced by the addition of modifiers or network forming oxides and their effects on the formation of polarons, or to the inherent property of polaron transport in IPG with low polaron concentration.

## Figures and Tables

**Table 1:** Batch compositions and selected properties of studied iron phosphate-based glasses containing B_2_O_3_ and HfO_2_.

Sample	Batch Composition (mol%) ^a^				Molar O/P Ratio	Molar Fe/P Ratio	N (Fe Ions) ×10^−21^/cm^−3^	*R* = *N*^−1/3^/Å
	B_2_O_3_	HfO_2_	P_2_O_5_	Fe_2_O_3_				
G–B0Hf0	-	-	60	40	3.5	0.67	9.63 ^b^	4.70 ^b^
	Series F							
F–B2Hf2	2	2	60	36	3.48	0.60	8.89 ^b^	4.83 ^b^
F–B4Hf4	4	4	60	32	3.47	0.53	8.00	5.00
F–B4Hf6	4	6	60	30	3.45	0.50	7.52	5.10
F–B8Hf8	8	8	60	24	3.43	0.40	6.16	5.46
	Series S							
S–B2Hf2	2	2	57.6	38.4	3.59	0.67	9.16	4.70
S–B4Hf4	4	4	55.2	36.2	3.68	0.67	9.26	4.76
S–B6Hf6	6	6	52.8	35.2	3.78	0.67	8.88	4.83
S–B4Hf8	4	8	52.8	35.2	3.76	0.67	11.64	4.41

^a^ from ref. [44] and ^b^ from ref. [53].

**Table 2:** Batch compositions and selected properties of studied iron phosphate-based glasses containing B_2_O_3_ and HfO_2_.

Samples	*σ*_DC_^a^/(Ω cm)^−1^ ± 0.5%	*E*_DC_/kJ mol^−1^ ± 0.5%	*σ*_0_*/(Ω cm)^−1^ K ± 0.5%	*C* ^b^	*exp*(−2*αR*)	*α*/Å^−1^
G–B0Hf0	1.00 × 10^−10^	62.4	3.14	0.23 ^c^	0.020	0.42
						
F–B2Hf2	8.71 × 10^−11^	62.2	3.08	0.58 ^c,d^	0.013	0.45
F–B4Hf4	5.01 × 10^−11^	62.9	2.96	0.17	0.017	0.40
F–B4Hf6	4.07 × 10^−11^	63.3	2.91	0.18	0.015	0.41
F–B8Hf8	1.12 × 10^−10^	60.0	2.77	0.36	0.008	0.45
						
S–B2Hf2	7.94 × 10^−10^	58.3	3.35	0.32 ^d^	0.027	0.38
S–B4Hf4	1.41 × 10^−10^	61.9	3.18	0.16 ^c^	0.029	0.37
S–B6Hf6	1.32 × 10^−9^	57.0	3.37	0.39	0.026	0.38
S–B4Hf8	1.35 × 10^−9^	56.9	3.33	0.38	0.006	0.58

^a^ Values at 30 °C, ^b^ *C* = Fe^2+^/Σ(Fe^2+^ + Fe^3+^) as obtained from Mössbauer spectra, ^c^ Mössbauer data reported from ref. [44], ^d^ In addition to obtaining Fe^2+^ concentration from this study, *N*_v_ is also calculated based on Mössbauer data reported in ref. [44], which show slightly higher values.

**Table 3:** Summary of parameters obtained from a detailed analysis of conductivity and permittivity spectra for all studied iron phosphate-based glasses containing B_2_O_3_ and HfO_2_.

Samples	*N*_v_ (Polarons) × 10^−21^/cm^−3^	*r*_p_/Å	log (*f*_shift_)	(Δε *T*) /K	<*r*_LOC_^2^(∞)>^1/2^/Å
F40	2.22 ^a^	1.89 ^a^	0	3910	2.25
					
F–B2Hf2 ^a^	3.73 ^a^	1.94 ^a^	+0.06	4564	1.93
F–B4Hf4	1.36	2.02	+0.01	4286	3.00
F–B4Hf6	1.35	2.06	−0.03	4103	2.94
F–B8Hf8	2.22	2.20	+0.03	4177	2.97
					
S–B2Hf2	3.08	1.90	+0.12	4994	2.15
S–B4Hf4	1.48	1.92	+0.11	5437	3.24
S–B6Hf6	3.46	1.95	+0.14	5742	2.18
S–B4Hf8	4.42	1.78	+0.10	4679	1.96

^a^ from ref. [53].

## Data Availability

The data presented in this study are available on request from the corresponding author.

## Supplementary Materials

The following supporting information can be downloaded at: https://www.mdpi.com/article/10.3390/nano12040639/s1, Figure S1. Mössbauer spectra of selected glass-(ceramics) samples from (a) F– and (b) S–series. Figure S2. Complex impedance plots @30 °C for all studied glass-(ceramic) samples from (a) F– and (b) S–series.
