# Supplementary material for: Electrical Transport in Iron Phosphate-Based Glass-(Ceramics): Insights into the Role of B2O3 and HfO2 from Model-Free Scaling Procedures
Source: Nanomaterials (Basel). 2022 Feb 14;12(4):639. doi: 10.3390/nano12040639 (PMC8878221; doi:10.3390/nano12040639)
Supplement: Supplementary file 1 [file nanomaterials-12-00639-s001.zip › nanomaterials-1515165-SI.pdf]

Supporting Information

# Electrical Transport in Iron Phosphate-Based Glass-(Ceramics): Insights into the Role of $B_2O_3$ and $HfO_2$ from Model-Free Scaling Procedures

**Arijeta Bafti [1], Shiro Kubuki [2], Hüseyin Ertap [3], Mustafa Yüksek [4], Mevlüt Karabulut [5], Andrea Moguš-Milanković [6] and Luka Pavić [6,*]**

[1] Faculty of Chemical Engineering and Technology, University of Zagreb, Marulićev trg 19, Zagreb 10000, Croatia; abafti@fkit.hr
[2] Department of Chemistry, Graduate School of Science, Tokyo Metropolitan University, 1-1, Minami-Osawa, Hachi-Oji, Tokyo 192-0397, Japan; kubuki@tmu.ac.jp
[3] Department of Physics, Kafkas University, Kars 36100, Turkey; huseyinertap@kafkas.edu.tr
[4] Electrical & Electronics Department, Faculty of Engineering and Natural Sciences, İskenderun Technical University, İskenderun 31200, Turkey; mustafa.yuksek@iste.edu.tr
[5] Department of Physics, Gebze Technical University, Gebze 41400, Turkey; mevlutk@gtu.edu.tr
[6] Ruđer Bošković Institute, 10000 Zagreb, Croatia; mogus@irb.hr
* Correspondence: lpavic@irb.hr

**Citation:** Bafti, A.; Kubuki, S.; Ertap, H.; Yüksek, M.; Karabulut, M.; Moguš-Milanković, A.; Pavić, L. Electrical Transport in Iron Phosphate-Based Glass-(Ceramics): Insights into the Role of $B_2O_3$ and $HfO_2$ from Model-Free Scaling Procedures. *Nanomaterials* **2022**, *12*, 639. https://doi.org/10.3390/nano12040639

Academic Editors: Xiang-Hua Zhang and Lothar Wondraczek

Received: 2 December 2021
Accepted: 11 February 2022
Published: 14 February 2022

**Publisher's Note:** MDPI stays neutral with regard to jurisdictional claims in published maps and institutional affiliations.

**Copyright:** © 2022 by the authors. Licensee MDPI, Basel, Switzerland. This article is an open access article distributed under the terms and conditions of the Creative Commons Attribution (CC BY) license (https://creativecommons.org/licenses/by/4.0/).

## Mossbauer spectroscopy

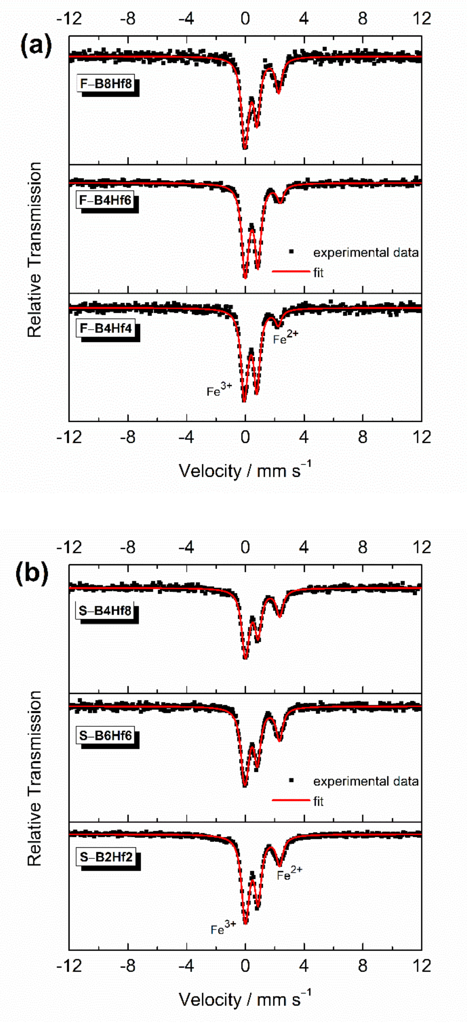


**Figure S1.** Mössbauer spectra of selected glass-(ceramics) samples from (**a**) F– and (**b**) S–series.

## Impedance spectroscopy

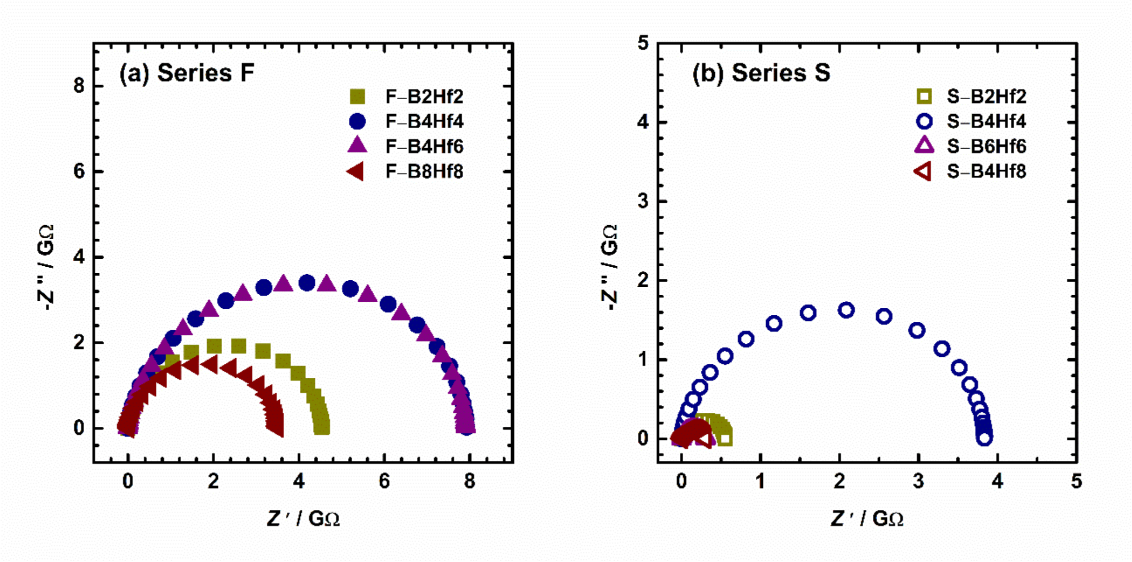


**Figure S2.** Complex impedance plots @30 °C for all studied glass-(ceramic) samples from (**a**) F– and (**b**) S–series.
